# When the tables are turned: The effects of the 2016 U.S. Presidential election on in-group favoritism and out-group hostility

**DOI:** 10.1371/journal.pone.0197848

**Published:** 2018-05-24

**Authors:** Burak Oc, Celia Moore, Michael R. Bashshur

**Affiliations:** 1 Department of Management and Organisations, University of Western Australia Business School, Crawley, Western Australia, Australia; 2 Department of Management and Technology, Bocconi University, Milan, Lombardy, Italy; 3 Lee Kong Chian School of Business, Singapore Management University, Singapore, Singapore; Coventry University, UNITED KINGDOM

## Abstract

The outcome of the 2016 U.S. Presidential election was a big surprise to many, as the majority of polls had predicted the opposite outcome. In this two-stage cross-sectional study, we focus on how Democrats and Republicans reacted to this electoral surprise and how these reactions might have influenced the way they allocated resources to each other in small groups. We find that, before the election, Republicans showed greater in-group favoritism than Democrats, who treated others equally, regardless of their political affiliation. We then show that Democrats experienced the election outcome as an ego shock and, in the week following the election, reported significantly higher levels of negative emotions and lower levels of self-esteem than Republicans. These reactions then predicted how individuals’ decided to allocate resources to others: after the election, Republicans no longer showed in-group favoritism, while Democrats showed out-group derogation. We find these decisions when the tables were turned can be partially explained by differences in participants’ state self-esteem.

## Introduction

On November 9, 2016, the *New York Times* announced, “Donald Trump’s Victory Is Met With Shock Across a Wide Political Divide” [1]. This headline captures two critical features of the 2016 U.S. presidential election. The first is how shocking the outcome was. Pollsters had estimated a 75% to 99% chance that Hillary Clinton would win [1,2], so Trump’s victory came as a surprise to most. The second is the degree of political polarization the 2016 election cycle highlighted. The divide between Democrats and Republicans in the U.S., in both voting trends [3] and political ideology [4], has increased dramatically in the last two decades, reaching fever pitch during this most recent presidential election. As a result, Democrats’ experienced Trump’s victory as a negative shock, while for Republicans the victory came as a positive surprise.

In this paper we explore how the shock of the unexpected outcome of this highly contested and important election, coupled with increasing political polarization nationally, affected how people in the U.S treat members of their political in-group and out-group. Specifically, we focus on how individuals who feel strong ties to either the Democratic or Republican Party treat each other when they control how resources are allocated, and how these patterns differed before and after the 2016 U.S. Presidential election. We observe that, prior to the election, Democrats treat others equally, regardless of their political affiliation, while Republicans show significant in-group favoritism (higher levels of generosity towards other Republicans). After the election, we demonstrate that these allocation patterns no longer held. Specifically, we argue that Democrats experienced the election as an ego shock (manifested in terms of low levels of state self-esteem), and this negative shock led to less generosity towards Republicans (out-group derogation). On the other hand, Republicans, experiencing the election as a positive shock, had less need to show in-group favoritism, and could be more generous to everyone.

### Before the election: Secure Democrats, threatened Republicans

A large body of research confirms that simply being a member of a group is sufficient to elicit more positive evaluations of fellow group members, as well as to like and favor them more than others [5]. This is because group membership provides individuals with a lens through which they construe their own reality and shapes their conscious and unconscious reactions toward in- and out-group members [6,7].

Political party affiliation is an important group membership for individuals [8], and represents a social identity that is highly salient during elections [9]. As part of increasing political polarization in the U.S., individuals tend to classify co-partisans as members of their in-group and opposing partisans as members of their out-group [4,10,11]. The resulting in-group bias means that individuals treat co-partisans better than opposing partisans, regardless of their political affiliation: Democrats favor Democrats, and Republicans favor Republicans (4).

This bias is especially evident when groups compete for scarce resources (e.g., money or political power). One of the earliest theories of intergroup threat, realistic group conflict theory (RGCT) [12, 13] suggests that in-group favoritism and out-group hostility are more likely to emerge when groups have conflicting goals. This is because the prospect of the out-group’s success threatens the in-group’s access to tangible resources, undermines their power over the out-group and thus the in-group’s potential to achieve its goals. As Sherif and Sherif [13] argued, such threats will impair inter-group relations by enhancing in-group friendship, exacerbating out-group hostility, and reinforcing in- and out-group distinctions. Although earlier studies of RGCT demonstrated that this in-group bias exists when the individuals involved are threatened directly, more recent work suggests that individuals also exhibit this bias when the interests of their group as a whole are threatened (even if they are threatened directly themselves) [14].

An election is an important type of resource competition, as its outcome creates a single winner, who then has greater control over resource distribution. Both RGCT and social identity explanations rooted in political partisanship predict in-group favoritism or out-group hostility, broadly, for members of both parties. However, drawing on theory about individuals’ fundamental desire to maintain a positive social identity under threat [15] helps us make more specific predictions about how an election outcome might affect resource allocations to political in-group and out-group members. On one hand, the prospect of losing a resource competition against an out-group represents a threat, which motivates individuals to protect this aspect of their social identity against the source of the threat. This motivation is driven largely by the desire to develop and maintain good relationships with other in-group members, and thus tends to manifest as in-group favoritism rather than as out-group derogation [16,17]. As Brewer [16] points out, that “it is easy to fear control by outsiders; perceived common threat from out-group increases in-group cohesion and loyalty” (p. 438).

On the other hand, individuals who are confident that their in-group will prevail in a resource competition do not have the same need to protect their social identity, and thus do not have the same motivation to demonstrate in-group favoritism. In fact, in the absence of threat, individuals may be indifferent towards out-group members or even show them sympathy, providing that in-group boundaries and intergroup distinctions are clearly defined (akin to “we have our ways, they have theirs”) [16]. Indeed, those who are confident of winning a resource competition may exhibit even more generosity towards out-groups. As Nadler [18] states, “members of higher status groups may give help to members of lower status groups not only out of caring and concern but also to maintain their social advantage” (p. 490). In other words, members of groups with confidence that they will maintain their power may implicitly assert their dominance to those in relatively less powerful groups by behaving more generously towards them.

These arguments underpin our primary prediction about how Republicans and Democrats treated co-partisans and opposing partisans before the election. Specifically, before the election, we expect Republicans to show in-group favoritism, as the polls heralding the impeding defeat of Trump in the election would be threatening and trigger the desire to reassert a positive social identity. In contrast, the polls prior to the election heightened Democrats’ confidence that they would continue to maintain control over the executive branch. As a result, they would have felt less threatened than Republicans, and thus have less need to assert a positive social identity, which would manifest in terms of less in-group favoritism. Their confidence that Clinton would win may have encouraged them to be more generous towards Republicans, as an assertion of their perceived political or power advantage. Taken together, we predict that, prior to the election, Republicans will favor other Republicans and thus allocate more resources to groups they believe to be more representative of their in-group, while Democrats will show less of this in-group favoritism (if any), and allocate resources more equally to others.

### After the election: Threatened Democrats, secure Republicans

Elections can be extremely distressing for those who supported losing candidates [19]. Specifically, supporters of losing candidates may not only feel rejected [20], anxious, angry [21], and extremely unhappy [8] but also threatened [22]. This was certainly the case for some of those who supported Clinton [23], who responded to the election using catastrophic language (e.g., This is a really tragic, unfortunate, terrible disaster”; “At a very base level, it’s terrifying to me”, cf. [24]).

One way to think about the extremely negative surprise this election outcome represented for Democrats is as an *ego-shock*. An ego-shock is caused by a major rejection, failure or traumatic event that ultimately shakes one’s self-esteem [25]. Indeed, people consistently report lower self-evaluations in losing situations than in winning situations [26]. Hence, as the supporters of the unexpected losing party, we hypothesize that Democrats will report more negative emotions, a larger ego-shock, and lower levels of self-esteem after the election, compared to Republicans.

One of the primary predictions of social identity theory is that threatened self-esteem promotes inter-group discrimination [27]. This is particularly likely when individuals identify strongly with the threatened social group [28], and the threat affects those individuals’ state self-esteem [17,29]. Both Branscombe and Wann [28] and Fein and Spencer [30] found that one of the outcomes of threat is out-group derogation. As Branscombe and Wann [28] explain, “when individuals with an important stake in a particular social identity were exposed to a social comparison that threatens the value of that identity, then the more their self-esteem is lowered [and] the more they would derogate the source of threat” (p. 642). Extrapolating from these findings, we hypothesize that Democrats, as the partisan losers of the election, will show more out-group derogation than Republicans, and allocate fewer resources to groups that contain more out-group members. In addition, we expect this effect to be amplified for those who identify strongly with the Democratic Party.

In contrast, individuals who do not experience a direct threat to a valued social identity will be less likely to show in-group favoritism or out-group derogation [31]. Hence, we expect Republicans, as the partisan winners of the election, will show neither in-group favoritism nor out-group derogation, and allocate resources equally to others irrespective of whether those individuals are members of their in-group or out-group. Furthermore, given that Republicans were not threatened by the election results, we do not expect group identification to moderate these effects for them [32].

Finally, we propose that Democrats’ and Republicans’ allocations following the election will be partly explained by their state self-esteem. Specifically, individuals with low state self-esteem tend to exhibit greater out-group derogation to enhance their self-image [17], while those with high state self-esteem are less likely to engage in out-group derogation [30]. Thus, we predict that lower levels of state self-esteem in the election’s aftermath will explain individuals’ lack of generosity towards out-groups after the election. As the more detrimentally affected party, we predict this effect to be particularly evident among Democrats.

## Materials and methods

In a two-stage cross-sectional study, we tested whether the allocations of self-identified Democrats and Republicans to members of their own and opposing political parties differed before and after the 2016 U.S. Presidential election. We conducted the first part of the study before the final primaries and national conventions of both parties (May 3–8, 2016), and the second part, using a new sample, in the week after the election (November 15–20, 2016). We pre-registered the second part of the study design (see https://osf.io/mujyg/). Experimental materials, data, and analysis code for both parts of the study reported in this article can be downloaded from the Open Science Framework at https://osf.io/vqy27/.

### Time 1: Before the election

#### Participants

We recruited 280 full-time working adults residing in the United States via Amazon Mechanical Turk. We determined this sample size a priori using a power analysis and considering possible exclusions. Specifically, an a priori analysis (calculating the sample size needed for a test of the main effects and the interaction of political party and group composition with a two-factorial design, numerator *df* = 2, number of groups = 6) using the G*Power software [33] indicated that a total sample size of 206 would be sufficient in order to reach a power of 90% to detect a medium effect size (*f* = 0.25) consistent with polarization effects reported in previous research [11]. Of these 280 participants, 38 did not feel strong ties to either the Democratic or Republican Party and 33 failed to correctly reply to questions designed to check whether they understood the game. These participants were not allowed to continue in the study. Furthermore, we excluded five participants who reported suspicion that other participants were fake.

We were left with 204 participants who felt meaningful ties to either the *Democratic* (*n* = 109) or *Republican* Party (*n* = 95) (48% female, average age 33.60 years, *SD* = 11.58). Of those, 174 self-identified as Caucasian, 12 as African American, 10 as Asian, and 8 as Hispanic. Thirty-six participants had post-graduate or graduate degrees, 83 had four-year college degrees and the remaining 85 had two-year college degrees or lower. With respect to annual income, 63.73% of participants reported their earnings as greater than $40,000 a year. Finally, participants earned $0.50 for their participation and had the opportunity to collect up to an additional $1.00 depending on the allocations they made in the study. Participants spent on average 12.56 minutes (*SD* = 3.41) completing the study.

#### Procedure and measures

Before providing participants with information about the design and procedure of the study, we asked them whether they identified more as a *Democrat*, a *Republican* or *felt no meaningful tie to either party*, and told them that this information would be shared with the other members of their group (we excluded participants who selected the “I feel no meaningful tie to either party” option).

We employed a multi-party dictator game [34], and informed participants that they would be part of a 4-person group (including themselves), and randomly assigned to be either a dictator, who would decide how much of a common resource ($1.00) they would keep for themselves and how much they would allocate to the other group members (to be divided equally amongst the three recipients). In reality, all participants were assigned to the dictator role, and the recipients were not real people. We formed groups differently for Republican and Democrat dictators in order to test whether they allocated a portion of the $1.00 common resource to others differently as a function of the group members’ political affiliations.

Specifically, we randomly assigned Republican participants to groups of either 1) three Republicans (i.e., all in-group members) (*n* = 31), 2) two Republicans and one Democrat (i.e., majority in-group members) (*n* = 32), or 3) one Republican and two Democrats (i.e., majority out-group members) (*n* = 32). In parallel, we randomly assigned Democrat participants to groups of either 1) three Democrats (i.e., all in-group members) (*n* = 38), 2) two Democrats and one Republican (i.e., majority in-group members) (*n* = 36), or 3) one Democrat and two Republicans (i.e., majority out-group members) (*n* = 35). Once provided with this information about the political party affiliation of their group members, we then asked participants to decide what proportion of the $1.00 they wanted to keep for themselves.

### Time 2: After the election

#### Participants

We recruited 280 full-time working adults residing in the United States via Amazon Mechanical Turk. In an effort to replicate the sample size and power of the first wave of data collection we used the same power analysis to determine the required sample size. Of these 280 participants, 31 did not feel strong ties to either the Democratic or Republican party and 40 failed to correctly reply to questions designed to check whether they understood the game. As in Time 1, these participants again were not allowed to continue in the study. Furthermore, we excluded three participants who reported suspicion that other participants were fake and six participants who failed the attention checks.

As in Time 1 (before the election), we report results for the 200 participants who understood the dynamics of the experimental game, did not report suspicion, provided complete responses and felt meaningful ties to either the *Democratic* (*n* = 107) or the *Republican* Party (*n* = 93) (49% female, average age 35.91 years, *SD* = 11.71). Of those, 148 self-identified as Caucasian, 18 as African American, 22 as Asian, and 12 as Hispanic. 41 participants had post-graduate and graduate degrees, 86 had four-year college degrees and the remaining 73 had two-year college degrees or lower. With respect to annual income, 62% of participants reported their earnings being greater than $40,000 a year. As in Time 1, we paid participants a $0.50 base rate and they had the opportunity to earn up to an additional $1.00 depending on the decisions they made in the study. The average time to complete the study was 12.40 minutes (*SD* = 4.87).

#### Procedure and measures

As in Time 1, we again randomly assigned Republican participants to groups of either 1) three Republicans (*n* = 29), 2) two Republicans and one Democrat (*n* = 32), or 3) one Republican and two Democrats (*n* = 32) and Democrat participants to groups of either 1) three Democrats (*n* = 36), 2) two Democrats and one Republican (*n* = 35), or 3) one Democrat and two Republicans (*n* = 36). The procedure was the same as Time 1, with several important exceptions. First, before receiving information about the dictator game, we asked participants several questions about their emotional and cognitive reactions to the result of United States presidential election. Second, in line with previous research on intergroup emotions [35], we asked participants to indicate on a 7-point scale (1 = *not at all*, 7 = *very much*) the extent to which they felt the following six positive emotions (*hope*, *pride*, *happiness*, *pleasantness*, *enthusiasm*, *gladness*) and six negative emotions (*hatred*, *hostility*, *anger*, *fear*, *paranoia*, *suspicion*) since the election results had been confirmed. Third, using four items of Campbell et al.’s [25] measure of ego shock (α = .94), we asked participants to rate on a 7-point scale (1 = *not at all*, 7 = *very much)* their endorsement of items such as, “Did you feel yourself “freeze up” or find yourself unable to act at any time immediately after the election results were announced?”. Fourth, in order to better capture the effect of the election results on participants’ self-esteem after the election results were announced, we asked participants to respond to Heatherton and Polivy’s [36] 20-item state self-esteem scale (SSES) (α = .94). This measure modified the Janis-Field Feelings of Inadequacy Scale [37] and was developed to capture state-dependent, momentary changes in one’s self-esteem or feelings about one’s abilities (as opposed to trait self-esteem, or general levels of self-esteem beyond the effects of situational factors). Previous studies have already provided empirical support for its factor structure as well as construct, content and discriminant validity [38]. Finally, we measured our participants’ level of identification with their political party on a 7-point scale (1 = *strongly disagree*, 7 = *strongly agree*) using Smith, Seger and Mackie’s [39] four-item identification measure (e.g., “I identify with other Republicans/Democrats”) (α = .93).

## Ethics statement

In both rounds of data collection, participants gave written informed consent before the study began and were paid for their participation. Full ethical approval for the study was received from Singapore Management University (Approval number: IRB-13-0028-A0034) and London Business School (Approval Number: REC91).

## Results and discussion

### Descriptive analyses

Table 1 provides descriptive statistics of the sample characteristics both in Time 1 (before the election) and in Time 2 (after the election). Our pre-registered hypotheses were that (1) there would be a difference in how self-identified Republicans and Democrats would allocate resources to others before and after the election, and that (2) their affective and cognitive reactions to the election outcome would help explain this difference. Building on this, we argued that Republicans would show more in-group favoritism before the election, but that after the election they would be less likely to allocate resources in a way that favors their in-group. On the other hand, we expected Democrats to allocate resources more equally to groups, regardless of the group’s composition, before the election, but that the negative shock of the election results would lead them to exhibit out-group derogation after the election, penalizing Republicans by allocating less to them. Moreover, we predicted this allocation pattern would be partly explained by low state self-esteem among Democrats after the election.

**Table 1:** Demographics of participants before and after the election.

	Before the election	After the election	Statistical difference
**Gender**	48% female	49% female	*p* = .93
**Age**	33.60 (*SD* = 11.58)	35.91 (*SD* = 11.71)	*p* = .18
**Race (in %)**			*p* = .15
Caucasian	85	74	
African American	6	9	
Asian	5	11	
Hispanic	4	6	
**Education (in %)**			*p* = .59
Post-graduate & graduate degrees	18	21	
Four year college degrees	41	43	
Two year college degrees and lower	42	37	
**Income greater than $40,000/year (in %)**	64	62	*p* = .15
**Political party affiliation (in %)**	53% Democrats	54% Democrats	*p* = .99

In order to make the inference using this two-wave cross sectional design that different allocation patterns before and after the election are a function of the election and not other causes, it is first necessary to demonstrate that the two samples are the same on all observable characteristics that might represent alternative explanations of our effects. The more similar the samples, the better the case is that differences in allocation patterns can be attributed to the election and not other causes. The composition of both samples did not differ in terms of gender (*X*^*2*^ (1) = .01, *p* = .93), age (*t* = -1.35, *p* = .18), race (*X*^*2*^ (3) = 9.56, *p* = .15), education (*X*^*2*^ (2) = 5.59, *p* = .59), income (*X*^*2*^ (8) = 12.08, *p* = .15), or political party affiliation (*X*^*2*^ (1) = .00, *p* = .99). In addition, we examined whether there were differences between our pre- and post-election samples of Democrats and Republicans. In fact, the composition of Democrats did not differ in terms of gender (*X*^*2*^ (1) = .01, *p* = .91), age (*t* = -1.56, *p* = .12), race (*X*^*2*^ (3) = 6.45, *p* = .10), education (*X*^*2*^ (2) = 8.59, *p* = .20), or income (*X*^*2*^ (8) = 4.82, *p* = .78). Similarly, Republicans did not differ in terms of gender (*X*^*2*^ (1) = .07, *p* = .79), age (*t* = -.36, *p* = .72), race (*X*^*2*^ (3) = 2.72, *p* = .44), education (*X*^*2*^ (2) = 7.20, *p* = .41), or income (*X*^*2*^ (8) = 10.11, *p* = .26).

We further examined the interaction between political party affiliation and time (pre- and post-election) to test whether Democrats were wealthier than Republicans (or vice versa) before versus after the election, information that is potentially important in interpreting our findings. Their interaction was non-significant (*F*(1, 399) = 0.14, *p* = .713, *η*_*p*_^*2*^ = .013) (suggesting the difference in our participants’ income as a function of political party affiliation across samples did not differ before and after election). Republicans reported higher incomes than Democrats, both before (*F*(1, 202) = 5.49, *p* = .020, *η*_*p*_^*2*^ = .083) and after the election (*F*(1, 197) = 3.33, *p* = .069, *η*_*p*_^*2*^ = .067). Different allocation patterns before and after the election can therefore not be explained by income changes within either political party affiliation before and after the election.

**Fig 1:**
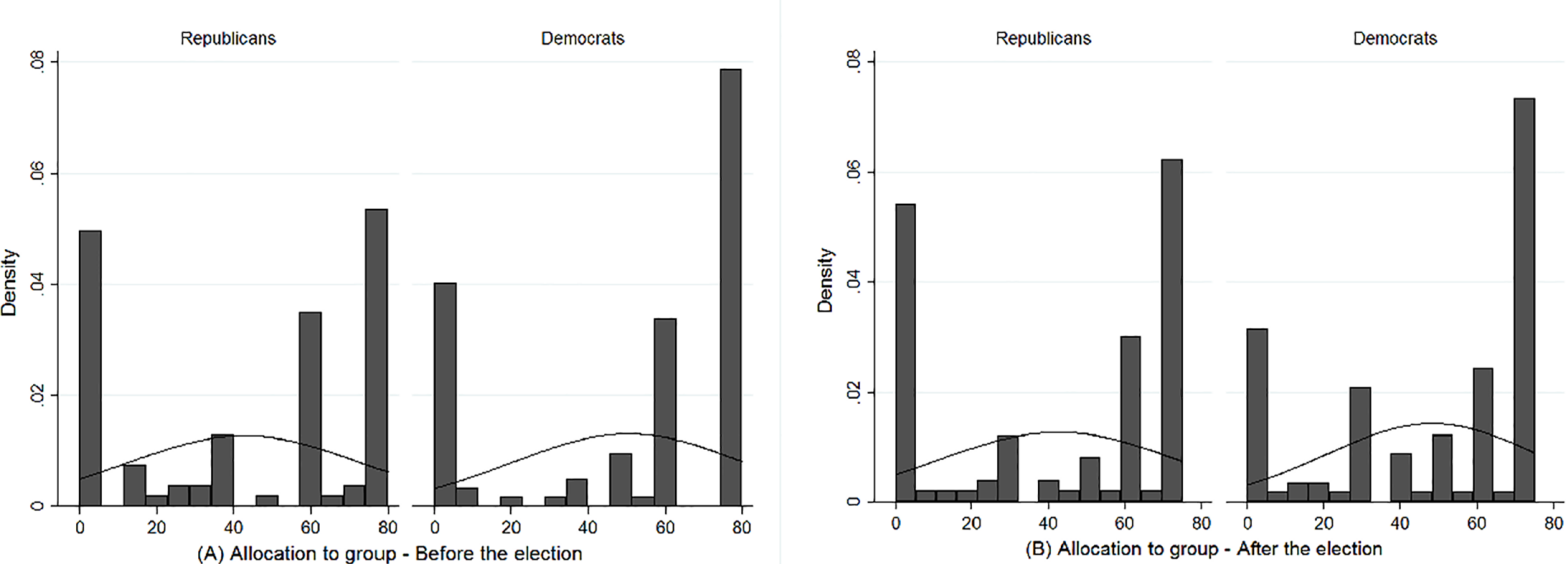
**The relative distribution of resource allocations to others (A) before and (B) after the election**.

Table 2 presents means, standard deviations, and zero-order Pearson correlations for the main study variables. Fig 1 displays the relative frequency distribution of allocations Republicans and Democrats made (A) before and (B) after the election. The allocations appear to be bipolar (the histogram is overlaid with an appropriately scaled normal density curve which is characterized by the mean and standard deviation of the allocations).

**Table 2:** Descriptive statistics and correlations.

	M	SD	(1)	(2)	(3)	(4)	(5)	(6)
(1) Time	.50	.50	-					
(2) Political party affiliation	.53	.50	.00	-				
(3) Group composition	.33	.47	.01	-.01	-			
(4) State self-esteem	3.77	.77	.	-.20	-.13	-		
(5) Party identification	5.18	1.29	.	.17	-.05	.05	-	
(6) Allocation to group	46.13	30.31	-.02	.11	-.12	.06	.08	-

*Note*. *N* = 404 for all variables except state self-esteem and party identification, which we only collected at time 2 and for which *N* = 200. Time dummy coded: 0 = Before the election, 1 = After the election, *Political party affiliation* dummy coded: 0 = Republican Party, 1 = Democratic Party. *Group composition* dummy coded: 0 = When recipients are majority in-group, 1 = When recipients are majority out-group. Correlations greater than |.10| are statistically significant at *p* < .05, two-tailed.

An analysis of variance using for the entire sample revealed a main effect for the political party affiliation of the participant, *F*(1, 392) = 4.81, *p* = .029, *η*_*p*_^*2*^ = .012, such that, on average, across both time periods and all group compositions, Republicans allocated less money to recipients (*M* = 42.55, *SD* = 31.26) than Democrats (*M* = 49.25, *SD* = 29.18). Additionally, group composition also had a main effect on the amount of money participants allocated to recipients, *F*(2, 392) = 5.47, *p* = .005, *η*_*p*_^*2*^ = .027, such that individuals allocated the most to others if their group was comprised of all in-group members (*M* = 52.91, *SD* = 31.26), less if their group included one out-group member (*M* = 44.55, *SD* = 30.80), and even less if their group included two out-group members (*M* = 40.99, *SD* = 30.61). The main effect for tine period was not significant, *F*(1, 392) = .14, *p* = .71, indicating that, overall, there was no change in the total amount allocated to group members across the time periods (*M*_Pre-election_ = 46.74, *SD* = 31.04 and *M*_Post-election_ = 45.52, *SD* = 29.62).

### Resource allocations before the election

Our pre-registered hypotheses were that the (1) allocations of self-identified Republicans and Democrats would change in the aftermath of the election, and that (2) these changes would be explained by participants’ affective and cognitive reactions to the election outcome. Building on this, we argued that, before the election, Republicans would show in-group favoritism, while Democrats would distribute allocations more equally. After the election, we expected Democrats to show out-group derogation, while Republicans would distribute allocations more equally. We additionally predicted that the negative shock of the election results and resulting drop in state self-esteem would partly explain why Democrats would penalize Republicans by allocating less to them after the election.

**Fig 2:**
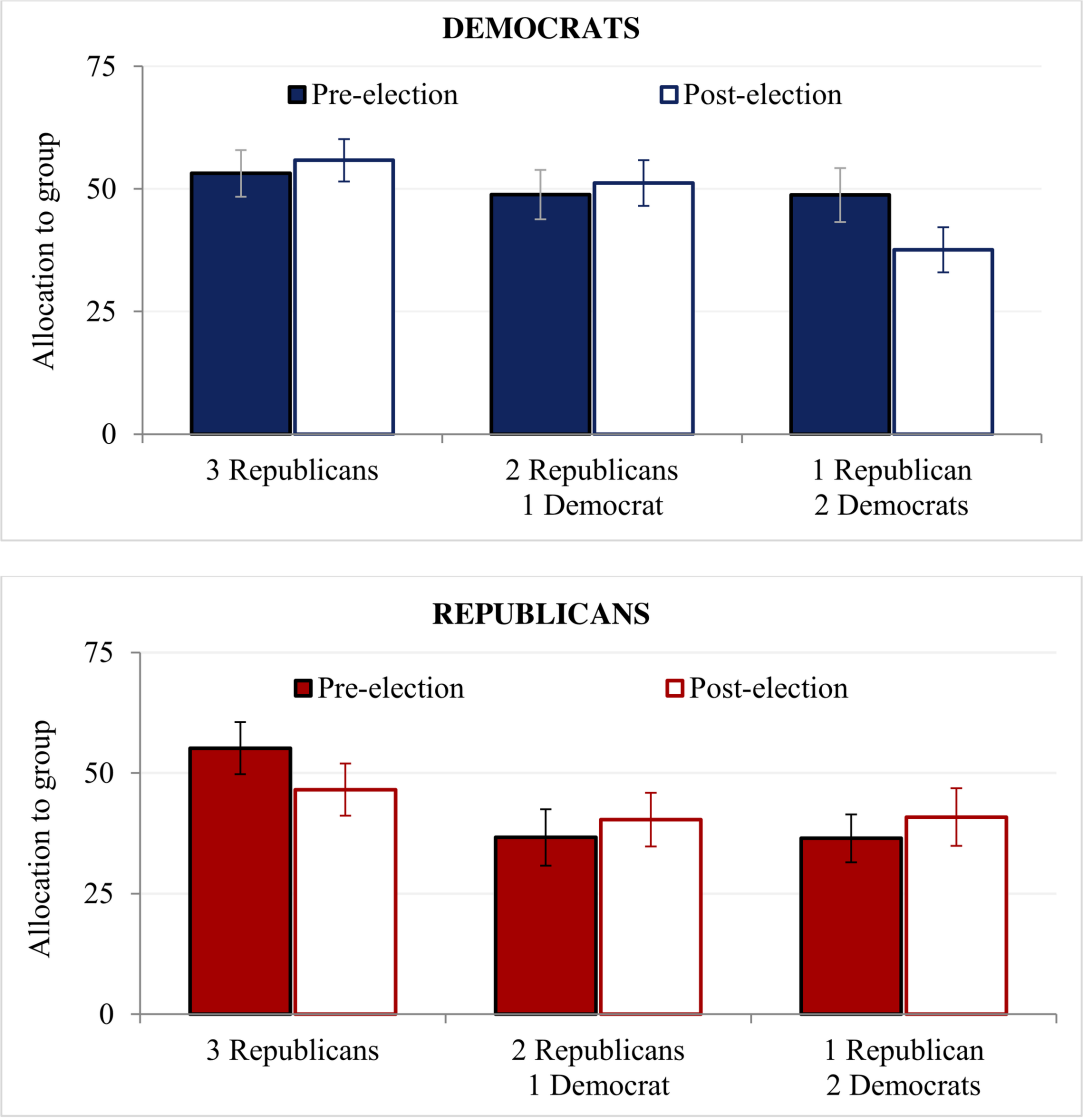
**Amount allocated by (A) Republicans and (B) Democrats before and after the election**.

To test whether, before the election, Republicans would show in-group favoritism while Democrats would distribute allocations more equally involves two specific contrasts. We find that Republicans showed significant in-group favoritism before the election, allocating significantly more to groups of all Republicans (*M* = 55.16, *SD* = 30.01) than to groups of 2 Republicans and 1 Democrat (*M* = 36.66, *SD* = 32.98), or groups of 2 Democrats and 1 Republican (*M* = 36.47, *SD* = 28.06), *F*(2, 392) = 4.04, *p* = .018, *η*_*p*_^*2*^ = .020 (see Republicans, pre-election in Fig 2). We also find that, before the election, Democrats make equal allocations to their groups, whatever the group composition. The allocations Democrats made to groups of all Democrats (*M* = 53.15, *SD* = 29.21) were not statistically different from the allocations they made to groups of 2 Democrats and 1 Republican (*M* = 48.83, *SD* = 30.23), or groups of 2 Republicans and 1 Democrat (*M* = 48.74, *SD* = 32.62), *F*(2, 392) = .26, *p* = .77, *η*_*p*_^*2*^ = .001 (see Democrats, pre-election in Fig 2).

### Resource allocations after the election

In contrast, after the election, Democrats penalized Republicans, allocating significantly less to groups of 2 Republicans and 1 Democrat (*M* = 37.56, *SD* = 27.52) than to groups of 2 Democrats and 1 Republican (*M* = 51.20, *SD* = 27.64), or groups of all Democrats (*M* = 55.83, *SD* = 25.98), *F*(2, 392) = 3.63, *p* = .027, *η*_*p*_^*2*^ = .018 (see Democrats, post-election in Fig 2). In contrast, Republicans’ in-group favoritism softened, and Republican allocators made equal allocations to groups, regardless of whether they were comprised of all Republicans (*M* = 46.55, *SD* = 29.15), 2 Republicans and 1 Democrat (*M* = 40.34, *SD* = 31.43), or 2 Democrats and 1 Republican (*M* = 40.87, *SD* = 33.71), *F*(2, 392) = .40, *p* = .67, *η*_*p*_^*2*^ = .002 (see Republicans, post-election in Fig 2).

### Emotional and cognitive reactions to the election

**Fig 3:**
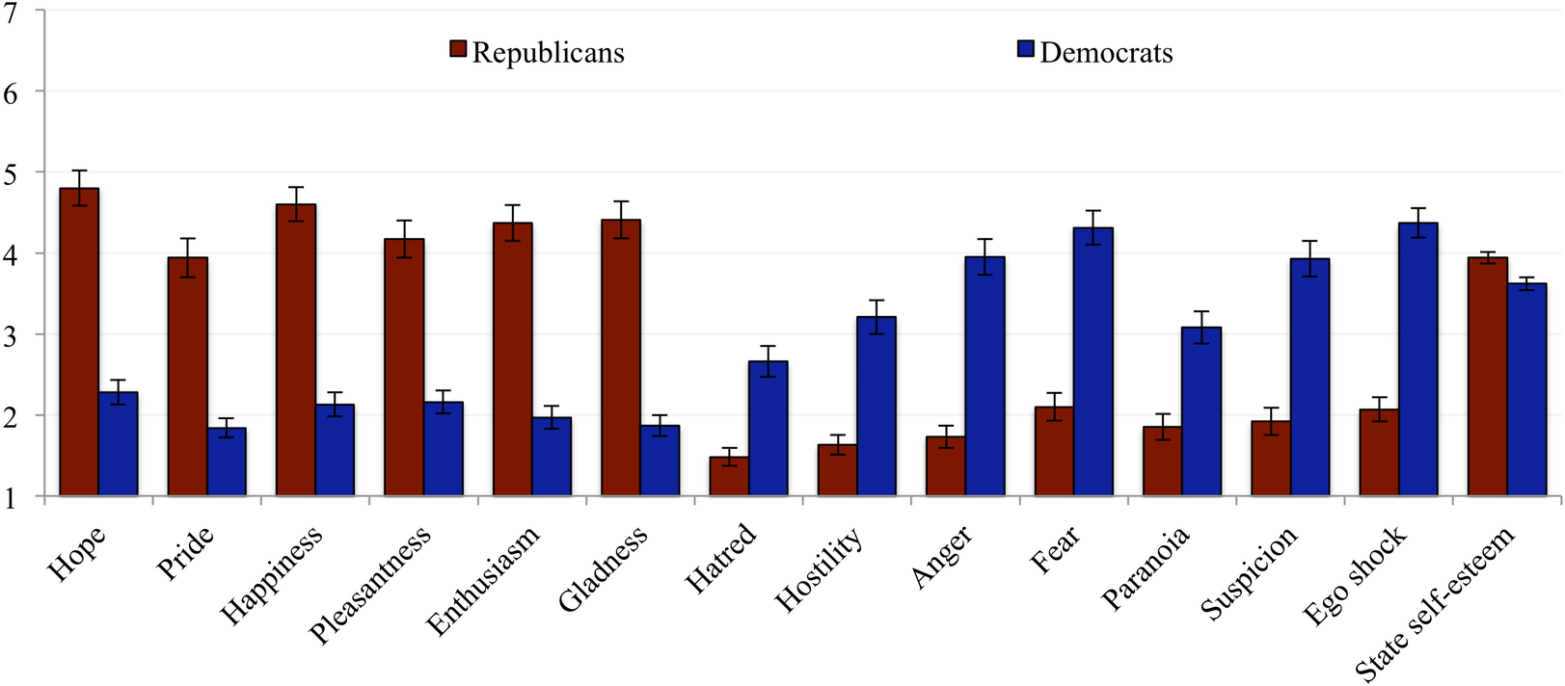
Participants’ affective and cognitive reactions after the election results were announced. Error bars indicate standard errors.

We argued that this difference in how Republicans and Democrats allocate resources to members of their in- and out-group before and after the election would be partially explained by participants’ emotional and cognitive reactions to the election results. Using the Time 2 data, we find an expected significant main effect of political party affiliation on each positive and negative emotion we asked participants to report: Democrats felt less *hope*, *pride*, *happiness*, *pleasantness*, *enthusiasm*, and *gladness* and felt more *hatred*, *hostility*, *anger*, *fear*, *paranoia*, and *suspicion* than Republicans after the election. Each of these differences were significant at *p*s < .001 (see Fig 3). Additionally, a two-tailed t-test confirmed that Democrats experienced the election as a greater ego shock than did Republicans (*M*_Democrats_ = 4.37, *SD*_Democrats_ = 1.84 vs. *M*_Republicans_ = 2.07, *SD*_Republicans_ = 1.40, *t*(198) = 9.79, *p* < .001, *d* = 1.41), and, relatedly (*r* = -.29, *p* < .001), reported lower levels of state self-esteem than Republicans (*M*_Democrats_ = 3.62, *SD*_Democrats_ = .84 vs. *M*_Republicans_ = 3.94, *SD*_Republicans_ = .65, *t*(198) = 2.91, *p* = .004, *d* = .43) after the election.

### Mediation by state self-esteem

We predicted that the effect of political party affiliation on resource allocations after the election would be partially mediated by state self-esteem. In other words, we predicted that being a Democrat would be associated with lower levels of state self-esteem after the election, which would motivate smaller allocations to others depending on whether they were co-partisans or opposing partisans. This is a moderated mediation hypothesis, which we tested using PROCESS Model 14 [40]. Specifically, we included political party affiliation as the independent variable, state self-esteem as the mediating variable, the amount allocated to others as the dependent variable, and the group composition as a variable moderating the relationship between state self-esteem and the allocation amount. For the sake of analytic parsimony, we created one dummy variable for the conditions where the majority of recipients are out-group members (i.e., two out-group members with one in-group member), so that conditions where the majority of recipients are in-group members (i.e., groups of all in-group members or one out-group member with two in-group members) represent the reference category. Results of the analysis are presented in Table 3.

**Table 3:** Conditional indirect effects of political affiliation on allocation to others via state self-esteem as a function of group composition.

	State self-esteem			Allocation to others		
	*b*	*SE*	*p*	*b*	*SE*	*p*
Constant	3.93	.08	.000	60.88	13.95	.000
Political party affiliation	-.31	.11	.004	7.42	4.17	.077
State self-esteem (SSE)				-4.18	3.46	.229
Group composition (GC)				-70.50	20.52	.001
SSE X GC				16.52	5.40	.003
*R*^2^	.04			.08		
Conditional indirect effect of political affiliation on allocation via state self-esteem:						95% CI
When majority in-group				1.31	1.25	[-.57, 4.60]
When majority out-group				-3.87	1.86	[-8.41, -.96]

*Note*. *N* = 200. *Political party affiliation* dummy coded: 0 = Republican Party, 1 = Democratic Party. *Group composition* dummy coded: 0 = When recipients are majority in-group, 1 = When recipients are majority out-group.

**Fig 4:**
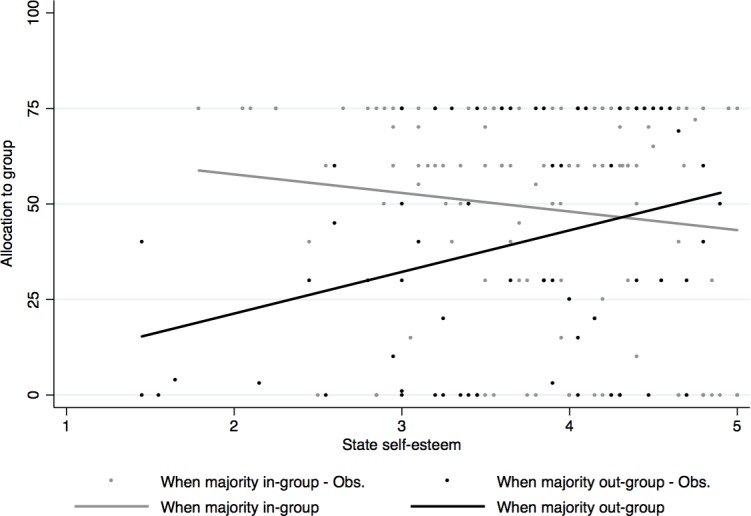
Two-way interaction between state self-esteem and group composition.

The mediator model showed that Democrats had significantly lower levels of self-esteem than Republicans (*b* = -.31, *p* = .004) after the election. The complete model shows the main effects for political party affiliation (Democrats, on average, allocating marginally more than Republicans, *b* = 7.42, *p* = .077), and majority out-group (smaller allocations to groups where the majority are members of the out-group, *b* = -70.50, *p* = .001). These effects were qualified by an interaction between state self-esteem and majority out-group (*b* = 16.52, *p* = .003). The results also confirmed a significant conditional indirect effect of political party affiliation on the amount of money allocated to others through state self-esteem when the majority of recipients were out-group members (*b* = -3.87, 95% confidence interval -8.41 to -.94), but not when the majority of recipients were in-group members (*b* = 1.31, 95% confidence interval -.62 to 4.46). When the majority of recipients were in-group members, state self-esteem did not mediate the effect of political party on allocations; however, when the majority of the recipients were out-group members, participants decreased their allocations as their self-esteem dropped, *t*(199) = 10.89, *p* = .009 (see Fig 4).

An additional analysis, using PROCESS Model 3 [40] to decompose the conditional effect of state self-esteem on allocations for different combinations of political party affiliation and group membership was only significant for Democrats making allocations to groups of majority Republicans (*b* = 11.05, *SE* = 5.10, 95% CI [.98, 21.10]). The conditional effect was not significant for Republicans making allocations to groups of majority Democrats (*b* = 12.73, *SE* = 8.69, 95% CI [-4.41, 29.88]).

### Moderation by strength of identification with one’s political party

We further predicted that this moderated mediated effect of political affiliation on the amount allocated after the election would be stronger for Democrats who identify strongly with their political party. In other words, we expected the indirect effect of political party affiliation on the amount allocated to others (through participants’ state self-esteem) would be moderated by not only the composition of the group, but also by the strength of the participant’s identification with his or her political party. Model 18 of Hayes’ PROCESS macro [40] tests the significance of this indirect effect as a function of dual moderators. It calculates confidence intervals for the conditional indirect effects of group composition and political party identification for each possible combination of these moderators’ values. The results of this model are presented in Table 4. Although the three-way interaction between state self-esteem, group composition and party identification was not significant (*b* = -2.04, *p* = .63), the conditional indirect effect for political party affiliation was present for groups with a majority of out-group members, when the strength of one’s party identification was 4.5 or higher.

**Table 4:** Conditional indirect effects of political affiliation on allocation to others via state self-esteem as a function of group composition and political party identification.

	State self-esteem			Allocation to others		
	*b*	*SE*	*p*	*b*	*SE*	*p*
Constant	3.93	.08	.000	112.83	58.13	.04
Political party affiliation	-.31	.11	.004	7.83	4.32	.07
State self-esteem (SSE)				-20.14	14.71	.17
Group composition (GC)				-103.20	85.67	.23
Party Identification (PI)				-9.59	10.67	.37
SSE x GC				5.63	16.08	.73
SSE x PI				28.06	22.49	.21
GC x PI				2.94	2.67	.27
SSE X GC x PI				-2.04	4.21	.63
*R*^2^	.04			.09		
Conditional indirect effect of political affiliation on allocation via state self-esteem:						95% CI
When majority in-group						
Party identification = -1SD				2.73	1.96	[-.22, 7.60]
Party identification = Mean				1.53	1.30	[-.47, 5.00]
Party identification = +1SD				.35	1.54	[-2.61, 3.71]
When majority out-group						
Party identification = -1SD				-3.59	2.49	[-9.54, .73]
Party identification = Mean				-3.95	1.91	[-8.60, -1.09]
Party identification = +1SD				-4.31	2.33	[-9.55, -.33]

*Note*. *N* = 200. *Political party affiliation* dummy coded: 0 = Republican Party, 1 = Democratic Party. *Group composition* dummy coded: 1 = When recipients are majority out-group, 0 = When recipients are majority in-group.

Finally, we wanted to explore whether the conditional effects of state self-esteem on the amount allocated as a function of strength of party identification differed as a function of individuals’ political party affiliations. We had predicted that the strength of identification would matter more for Democrats after the election than for Republicans.

For ease of interpretation, we ran two separate PROCESS Model 3 analyses, one for Democrats and one for Republicans, to determine if the conditional effect of state self-esteem on allocations as a function of group composition and party identification differed for Democrats and Republicans. For Republicans, allocations did not vary as a function of the strength of their identification with their political party and state self-esteem. However, for Democrats, the conditional effect of state self-esteem on allocations to groups with majority Republicans only held for those at the high end (5.25 and higher) of party identification. These findings confirm that Democrats’ social identities played an important role in how the negative shock of the election ultimately lessened their generosity towards Republicans.

## Conclusions

In this research, we provide empirical evidence that how Democrats and Republicans treat others was different before and after the 2016 United States presidential election as a function of political party affiliation. Before the election, Democrats, confident Clinton would be elected, allocated resources equally to others across groups, regardless of whether group members were Democrats or Republicans. In contrast, Republicans, who had reasonable concerns that Trump would lose, showed significant in-group favoritism and allocated more resources to groups where the majority of recipients were in-group members (i.e., Republicans).

After the election, the tables were turned. Democrats reported experiencing significantly higher levels of negative emotions and significantly lower levels of positive emotions than Republicans, as well as significantly higher levels of ego shock and lower levels of state self-esteem. What’s more, after the election, Democrats allocated resources to others differently than they did before the election, demonstrating significant out-group derogation and allocating fewer resources to groups where the majority of recipients were Republicans. Conversely, after the election Republicans behaved more like Democrats had behaved prior to the election, giving equally to others, regardless of their political party affiliation.

We explored the role of state self-esteem as a potential underlying mechanism explaining these findings, in particular focusing on resource allocations after the election. Indeed, individuals (and Democrats in particular) allocated fewer resources to others in groups where the majority of recipients were out-group members as a function of their state self-esteem, suggesting avarice towards the out-group was a reaction to the negative shock of the election. Overall, we believe our findings provide evidence that unexpected outcomes, or shocks, can influence how individuals treat members of their in-group and out-group. Self-esteem interacted with in-group identification and the presence or absence of out-group members to help explain the level of generosity or avarice expressed.

That being said, our results indicate that state self-esteem only partially mediated the effects of election results (or political party affiliation) on allocation to others. Although the direct effect of political party affiliation remained significant, state self-esteem accounted for approximately 18 percent of the direct effect on allocations to others when recipients are majority in-group and 52 percent when recipients are majority out-group, suggesting there are alternative mechanisms that also drive the effects of election results on allocations to others. One additional mechanism that seems likely is the negative affective reaction Democrats experienced in response to the result. Indeed, some Clinton supporters report being afraid or traumatized as a result of the election outcome [24]. These negative emotions (fear, anger, etc.) could have (along with state self-esteem) contributed to increased hostility towards out-groups on the part of Democrats [41].

We also acknowledge that employing a two-stage cross-sectional, between-person design rather than a longitudinal, within-person design with the same participants before and after the election, somewhat compromises our ability to make strong inferences about the extent to which the election outcome actually changed how Democrats and Republicans allocated resources to others. Nevertheless, given that participant demographics remained constant across both time periods, we are fairly confident about using our findings to argue that the election outcome contributed to the extent to which Democrats and Republicans showed in-group favoritism and out-group hostility before and after the election.

Finally, by necessity, we focused on one particular type of shock in this paper, and used a somewhat artificial setting in which individuals made decisions about resources independent of context other than political party affiliation. Future research might fruitfully examine the influence of other type of shocks (e.g., peer rejection, failure) in broader contexts requiring more interaction (e.g., social dilemmas, [42]) to determine if the effect of ego shocks on self-esteem persists in these types of settings. However, the negative affect Democrats feel towards Republicans does not seem to be waning [43], suggesting that these effects might be longer lasting than anyone might have expected.
